# Generalizations of the genomic rank distance to indels

**DOI:** 10.1093/bioinformatics/btad087

**Published:** 2023-02-15

**Authors:** João Paulo Pereira Zanetti, Lucas Peres Oliveira, Leonid Chindelevitch, João Meidanis

**Affiliations:** Institute of Computing, University of Campinas, Campinas, Brazil; Institute of Computing, University of Campinas, Campinas, Brazil; MRC Centre for Global Infectious Disease Analysis, School of Public Health, Imperial College, London, UK; Institute of Computing, University of Campinas, Campinas, Brazil

## Abstract

**Motivation:**

The rank distance model represents genome rearrangements in multi-chromosomal genomes as matrix operations, which allows the reconstruction of parsimonious histories of evolution by rearrangements. We seek to generalize this model by allowing for genomes with different gene content, to accommodate a broader range of biological contexts. We approach this generalization by using a matrix representation of genomes. This leads to simple distance formulas and sorting algorithms for genomes with different gene contents, but without duplications.

**Results:**

We generalize the rank distance to genomes with different gene content in two different ways. The first approach adds insertions, deletions and the substitution of a single extremity to the basic operations. We show how to efficiently compute this distance. To avoid genomes with incomplete markers, our alternative distance, the rank-indel distance, only uses insertions and deletions of entire chromosomes. We construct phylogenetic trees with our distances and the DCJ-Indel distance for simulated data and real prokaryotic genomes, and compare them against reference trees. For simulated data, our distances outperform the DCJ-Indel distance using the Quartet metric as baseline. This suggests that rank distances are more robust for comparing distantly related species. For real prokaryotic genomes, all rearrangement-based distances yield phylogenetic trees that are topologically distant from the reference (65% similarity with Quartet metric), but are able to cluster related species within their respective clades and distinguish the *Shigella* strains as the farthest relative of the *Escherichia coli* strains, a feature not seen in the reference tree.

**Availability and implementation:**

Code and instructions are available at https://github.com/meidanis-lab/rank-indel.

**Supplementary information:**

Supplementary data are available at *Bioinformatics* online.

## 1 Introduction

In the context of genome comparison, one can view a genome as a collection of contiguous, conserved segments arranged in linear and/or circular chromosomes. These segments can be genes or long, continuous stretches of very similar DNA sequences. Here, we use the term ‘markers’ to mean either of these cases. In this abstraction, we pay no attention to point mutations, and focus instead on larger rearrangements, changing the order of segments with respect to one another. A rearrangement event commonly seen in bacterial genomes is known as chromosomal inversion, which is a major driver for their adaptation to a changing environment (Noureen *et al.*, 2019). Another example of such rearrangements, known as chromosomal translocation, occurs when a portion of one chromosome is interchanged with a portion of a different chromosome, and is a hallmark of cancer (Hogenbirk *et al.*, 2016). Since such events are much rarer than nucleotide substitutions, they have the potential to serve as good indicators of how evolution unfolded in a larger time span.

In simpler models of genome rearrangement, the operations only move genomic segments around, without creating or destroying markers. However, to better reflect genome evolution, it is desirable to include operations that alter the content of the genome. For example, we may consider operations that add contiguous segments to the genome, called *insertions*, and operations that remove contiguous segments from the genome, called *deletions*. In general, we call these two types of operation *indels*. To the best of our knowledge, the work on including indels in genome rearrangement models has so far been limited to the inversion distance (Hannenhalli and Pevzner, 1999) for unichromosomal genomes, and the Double-Cut-and-Join (DCJ) distance (Yancopoulos *et al.*, 2005) on multi-chromosomal genomes (Braga, 2013; Paten *et al.*, 2014).

El-Mabrouk (2001) first studied the problem of sorting by inversions and indels, developing an exact algorithm for the cases where there were only insertions or deletions, but not both. Yancopoulos and Friedberg (2009) proposed extending the DCJ model to account for insertions and deletions, and Braga *et al.* (2011a) presented a linear-time algorithm for the DCJ-Indel problem. Later, Compeau (2013) used a different approach, looking at indels as DCJ operations themselves, and arrived at a simpler DCJ-Indel distance formula and sorting algorithm. Another extension of the DCJ model by Braga *et al.* (2011b) comes from adding a more powerful operation: a *substitution* of a genome segment for another. The development of DCJ-Indel also led to advancements on the inversion-indel distance, by Willing *et al.* (2013). The aforementioned results rely on the assumption that the genomes under comparison have the same set of unique markers. Shao *et al.* (2015) further extended the DCJ distance by allowing for duplicate markers. This generalized version of the genomic distance problem becomes NP-hard, and they give an Integer Linear Programming (ILP) formulation to compute the distance. However, their approach is limited to genomes that have equal numbers of duplicates of any marker. Later, Bohnenkämper *et al.* (2021) proposed an ILP formulation to compute the DCJ distance for natural genomes—in which any marker may occur an arbitrary number of times—which, very recently, was improved by Rubert *et al.* (2021).

One of the main concerns with the addition of indel operations to a genomic distance is respecting the triangle inequality. When indels have a constant cost, the triangle inequality is easily violated. Yancopoulos and Friedberg (2009) call this violation ‘the free lunch problem’. Their suggestion to deal with this problem is to add a surcharge to the cost of an indel, based on the adjacency graph. Braga *et al.* (2011a) dealt with the violation of the triangle inequality by adding a simpler surcharge after the computation of their DCJ-indel distance. In addition, Braga *et al.* (2011c) defined a framework to assign variable costs to indels, a linear function of the number of markers inserted or deleted, and showed that it is equivalent to the *a posteriori* surcharge.

Another point worth considering involves computing median genomes, which are important in the context of constructing ancestral genomes in a given phylogeny (Tannier *et al.*, 2009). For the DCJ distance, finding a median of three genomes is NP-hard (Tannier *et al.*, 2009). For the rank distance, however, this problem is still open, and there are polynomial-time algorithms to compute the rank median of three matrices, some of them running in cubic time when the inputs are genomic (Meidanis and Chindelevitch, 2021). Although this algorithm sometimes yields non-genomic matrices, it is an encouraging step in a field with mostly negative results.

In this article, we explore the addition of indels to the rank distance model, which was initially developed for same-content genomes (Zanetti *et al.*, 2016). In this model, genomes are represented as matrices, and the distance between two genomes is the rank of their difference. We expect this model to have a natural extension to genomes with unequal content, leading to simple formulas and algorithms. Unlike the DCJ distance, the rank distance, with the proposed extension to the matrix representation of genomes, naturally offers an indel mechanism with weights that avoid the free lunch problem.

A summary of these results appear in Table 1.

**Table 1.** Computational complexities for the best known algorithms in genome comparison, by means of the DCJ, rank and rank-indel distances, with respect to the number *n* of markers

	DCJ					
	Same content		Indels		Repetitions	
Distance	*O*(*n*)	Yancopoulos *et al.* (2005)	*O*(*n*)	Braga *et al.* (2010, 2011a)	ILP	Bohnenkämper *et al.* (2021)
Scenarios	Output size	Braga and Stoye (2010)	Characterization	Compeau (2013)	Open	—
Median	NP-hard	Tannier *et al.* (2009)	NP-hard^a^	—	NP-hard	—

	Rank				
	Same content		Indels		Repetitions
Distance	*O*(*n*)	Feijão and Meidanis (2013)	*O*(*n*)	This article	Open
Scenarios	Output size	Zanetti *et al.* (2019)	Open	—	Open
Median	$O(n^{3})$ ^b^	Meidanis and Chindelevitch (2021)	Open	—	Open

a By straightforward reduction.

b Sometimes not genomic.

The rest of this article is organized as follows. Section 2 presents the background on the rank distance and defines the representation of genomes that do not necessarily have all the markers being considered. In Section 3, we expand the rank distance to encompass genomes with different genomic content. In Section 4, we present a different approach for adding indels to the rank distance model. Section 5 describes our experiments, and Section 6 presents our conclusions.

## 2 Definitions

### 2.1 Markers, genomes and matrices

We begin our definitions with the notion of a *marker*, which can be a contiguous DNA stretch that is conserved in all genomes where it appears, or a gene, or an operon, or any other conserved marker of interest. This will be our building block in constructing genomes.

Let G be a set of markers. Each marker g∈G has two extremities: a head *g_h_* and a tail *g_t_*. The set


$$V(G)=\{g_{h},g_{t}|g\in G\}$$

contains all extremities associated to G. We will fix a 1–1 mapping identifying V(G) with the canonical basis $\left\{e_{1},e_{2},\ldots ,e_{2n}\right\}$ of $R^{2n}$, where n=|G| and *e_i_* is the 2n×1 column vector whose *i*th entry is 1 and all others are 0. Since this mapping is fixed, we will use the same letter and type font to denote both an extremity *x* and its corresponding column vector.

A genome *A* over G consists of a set V(A)⊆V(G) of extremities and a set *E*(*A*) of *adjacencies*, which are unordered pairs of distinct extremities from *V*(*A*), with the extra restriction that each extremity in *V*(*A*) can belong to at most one adjacency. Note that a genome does not necessarily contain all the extremities from all the markers in G. We do not even require that $g_{t}\in V(A)$ if $g_{h}\in V(A)$, and *vice versa*. The reason for that will become clear in Section 3.

If a pair {*x*, *y*} belongs to *E*(*A*), we say that *x* and *y* are *adjacent* in genome *A*. From the definitions, we see that each extremity x∈V(A) has to either be adjacent to exactly one other extremity, or be a *free end*, i.e. an extremity not adjacent to any other. This happens near the end of a linear chromosome. Circular chromosomes do not have free ends. Therefore, this representation contemplates both circular and linear chromosomes. In addition, extremities from V(G) that do not belong to *V*(*A*) will be called *A-null*, because they will correspond to null rows and columns in the matrix for *A*, as we will see shortly.

**Fig. 1.**
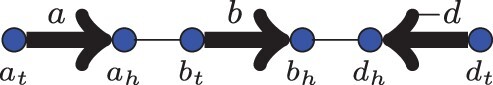
Genome *A* with extremity set $V(A)=\{a_{h},b_{h},d_{h},a_{t},b_{t},d_{t}\}$ and adjacency set $E(A)=\{\{a_{h},b_{t}\},\{b_{h},d_{h}\}\}$

For example, let G={a,b,c,d}, and let *A* be a genome with extremity set $V(A)=\{a_{h},b_{h},d_{h},a_{t},b_{t},d_{t}\}$, and adjacency set $E(A)=\{\{a_{h},b_{t}\},\{b_{h},d_{h}\}\}$. Genome *A* is illustrated in Figure 1.

Given that extremities are identified with column vectors of $R^{2n}$, we may view genomes as matrices as follows. Using the same letter and typeface *A* to represent the matrix associated to the genome *A*, we will define:


Ax={y, when {x,y}∈E(A),x, when x is a free end in V(A),0, when x∈V(A).

This formula unambiguously defines *A*, since it specifies the image under *A* of a basis of $R^{2n}$. As an example, the matrix representation for the genome *A* in Figure 1 is:


$$\begin{array}{cc} & \begin{array}{cccccccc} a_{t} & a_{h} & b_{t} & b_{h} & c_{t} & c_{h} & d_{t} & d_{h} \end{array}\\ \begin{array}{c} a_{t}\\ a_{h}\\ b_{t}\\ b_{h}\\ c_{t}\\ c_{h}\\ d_{t}\\ d_{h} \end{array} & \left[\begin{array}{cccccccc} 1 & 0 & 0 & 0 & 0 & 0 & 0 & 0\\ 0 & 0 & 1 & 0 & 0 & 0 & 0 & 0\\ 0 & 1 & 0 & 0 & 0 & 0 & 0 & 0\\ 0 & 0 & 0 & 0 & 0 & 0 & 0 & 1\\ 0 & 0 & 0 & 0 & 0 & 0 & 0 & 0\\ 0 & 0 & 0 & 0 & 0 & 0 & 0 & 0\\ 0 & 0 & 0 & 0 & 0 & 0 & 1 & 0\\ 0 & 0 & 0 & 1 & 0 & 0 & 0 & 0 \end{array}\right] \end{array},$$

where $a_{t}=e_{1},\;a_{h}=e_{2}$,…, $d_{h}=e_{8}$. A matrix that can be obtained from a genome in this fashion will be called a *genomic matrix*. It is easy to see that a square binary matrix $A\in {\left\{0,1\right\}}^{2n\times 2n}$ is genomic if and only if $A^{T}=A$ and *A*^2^ is a diagonal matrix with 0’s and 1’s on the diagonal; indeed, the 1 entries on the diagonal of *A*^2^correspond to the extremities present in *V*(*A*).

### 2.2 Rank distance

Let *A* and *B* be two genomic matrices. We can define a distance between them as follows:


$$d_{r}(A,B)=r(B-A),$$

where *r*(*X*) denotes the rank of matrix *X*. For invertible genome matrices *A* and *B*, which do not have zero rows or columns and therefore include all the extremities, this definition generalizes the rank distance of Zanetti *et al.* (2016). This distance satisfies the required properties for a metric:

-
$d_{r}(A,B)=0\Leftrightarrow A=B$

-
$d_{r}(A,B)=d_{r}(B,A)$

-
$d_{r}(A,C)\leq d_{r}(A,B)+d_{r}(B,C).$

**Fig. 2.**
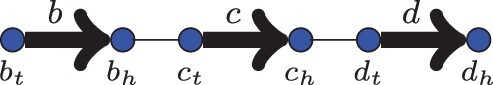
Chromosomal representation of a genome *B* with $V(B)=\{b_{h},c_{h},d_{h},b_{t},c_{t},d_{t}\}$ and adjacencies $\left\{\{b_{h},c_{t}\},\{c_{h},d_{t}\}\right\}$

For example, consider the genome *A* defined above, and let *B* be the following genome, illustrated in Figure 2:


$$B=\left[\begin{array}{cccccccc} 0 & 0 & 0 & 0 & 0 & 0 & 0 & 0\\ 0 & 0 & 0 & 0 & 0 & 0 & 0 & 0\\ 0 & 0 & 1 & 0 & 0 & 0 & 0 & 0\\ 0 & 0 & 0 & 0 & 1 & 0 & 0 & 0\\ 0 & 0 & 0 & 1 & 0 & 0 & 0 & 0\\ 0 & 0 & 0 & 0 & 0 & 0 & 1 & 0\\ 0 & 0 & 0 & 0 & 0 & 1 & 0 & 0\\ 0 & 0 & 0 & 0 & 0 & 0 & 0 & 1 \end{array}\right].$$

Having matrices for both *A* and *B* on hand, we can compute their difference:


$$B-A=\left[\begin{array}{cccccccc} -1 & 0 & 0 & 0 & 0 & 0 & 0 & 0\\ 0 & 0 & -1 & 0 & 0 & 0 & 0 & 0\\ 0 & -1 & 1 & 0 & 0 & 0 & 0 & 0\\ 0 & 0 & 0 & 0 & 1 & 0 & 0 & -1\\ 0 & 0 & 0 & 1 & 0 & 0 & 0 & 0\\ 0 & 0 & 0 & 0 & 0 & 0 & 1 & 0\\ 0 & 0 & 0 & 0 & 0 & 1 & -1 & 0\\ 0 & 0 & 0 & -1 & 0 & 0 & 0 & 1 \end{array}\right].$$

Thus, we have the distance $d_{r}(A,B)=r(B-A)=8$. However, computing the rank of the matrix *B–A* directly is not the most computationally efficient way to compute the rank distance. In Section 3.1, we will see how to do that in *O*(*n*) time.

### 2.3 Augmented breakpoint graph

To prepare for the addition of indels to the rank distance model, we defined genomes so that they do not necessarily have the same gene content. We use a structure called the *augmented breakpoint graph*, analogous to the regular breakpoint graph, but, following Compeau (2013), with different classifications for path endpoints.

The nodes of the augmented breakpoint graph *BG*(*A*, *B*) of *A* and *B* are the extremities of the set V(G)⊇V(A)∪V(B), and two nodes *x* and *y* are adjacent in *BG*(*A*, *B*) if they are adjacent in either *A* or *B*. For visual convenience, we represent the adjacencies from *A* with black solid edges and those from *B* with grey dashed edges. As in the regular breakpoint graph, all components are either paths or cycles. Sometimes, we refer to them as a *k*-path or a *k*-cycle when we want to emphasize that *k* is the number of edges in the path or the cycle.

In the augmented breakpoint graph, all nodes with degree 2 are necessarily in V(A)∩V(B), because they are parts of adjacencies in both genomes. On the other hand, a node *x* with degree 1 is a path endpoint, and at least one of the following cases applies:

- *x* is a free end in *A*: *Ax*=*x*,
- *x* is a free end in *B*: *Bx*=*x*,
- *x* is *A*-null: *Ax*=0,
- *x* is *B*-null: *Bx*=0.

When a path has at least one edge, then it has exactly two distinct end nodes. For each of these two nodes at the ends of the path, exactly one of the cases above apply. When both endpoints are free ends, we call the path *proper*. We say a path is *A-null* (*B-null*) when one of its ends is a free end, and the other is an *A*-null (*B*-null) node. When a path has two distinct *A*-null (*B*-null) ends, we call the path *AA-null* (*BB-null*). In the case where one end is *A*-null and the other is *B*-null, the path is called *AB-null*.

Finally, when a node *x* has degree zero in *BG*(*A*, *B*), exactly two of the previous cases apply, leading to four possibilities:

- When *x* is a free end in both *A* and *B*, it forms a proper path;
- When *x* is a free end in *A* and *B*-null, it forms a *B*-null path;
- When *x* is *A*-null and a free end in *B*, it forms an *A*-null path;
- Finally, when *x* is null in both *A* and *B*, the ‘natural’ definition would be to consider it an *AB*-null path. However, as we will see in Section 4, for the rank-indel distance it makes more sense to consider this path a proper path. For the rank distance, it makes no difference to consider it as either a proper path or an *AB*-null path. We choose to adopt the convention that it is a proper path, to accommodate both versions of the distance.

**Fig. 3.**
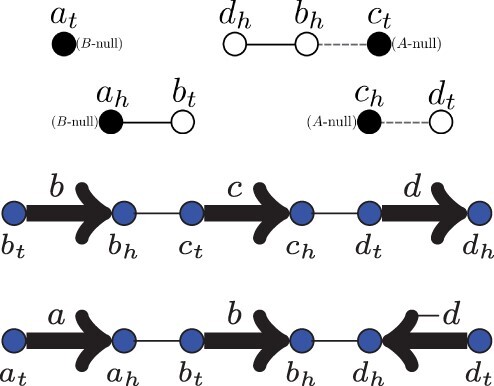
Augmented breakpoint graph *BG*(*A*, *B*). Black solid edges are adjacencies from *A*, gray dashed edges are from *B*. White nodes are extremities in both *V*(*A*) and *V*(*B*). Black nodes are either *A*-null or *B*-null, as specified besides them. The components are two *A*-null paths and two *B*-null paths. For convenience, we reproduce here the chromosomal representation of both genomes, *A* on top and *B* in the bottom

As an example, Figure 3 is the augmented breakpoint graph *BG*(*A*, *B*) of the genomes *A* and *B* seen earlier.

Given two genomes *A* and *B*, we will define some statistics for *BG*(*A*, *B*). We will use *c*(*A*, *B*) and *p*(*A*, *B*) to denote, respectively, the number of cycles and paths in *BG*(*A*, *B*). The number of paths can be further decomposed as the sum of the number of paths of each type: $p_{0}(A,B)$ is the number of proper paths in *BG*(*A*, *B*), while $p_{A}(A,B),\;p_{B}(A,B),\;p_{AA}(A,B),\;p_{BB}(A,B)$ and $p_{AB}(A,B)$ are the number of *A*-null, *B*-null, *AA*-null, *BB*-null and *AB*-null paths, respectively.

## 3 Rank distance in the presence of indels

In this section, we discuss the rank distance of genomes with possibly different marker content. First, in Section 3.1, we provide a linear-time algorithm to compute the rank distance. Then, in Section 3.2, we define the most concise set of operations needed to transform one genome into another. Finally, in Section 3.3, we show how to use these operations to optimally sort genomes.

### 3.1 Efficient computation of the rank distance

Algorithm 1 implements the ideas of Theorem 8 in boxedSupplementary Appendix SA and runs in *O*(*n*) time, efficiently computing $d_{r}(A,B)=r(B-A)$. It is a Breadth-First Search traversing *BG*(*A*, *B*) that additionally computes a score *s* for each component, equal to the difference between the number of *A*-null and *B*-null extremities in it. Note that extremities *i* such that A[i]>0 and B[i]>0 contribute zero to the score. A score of zero means the component has the same number of *A*-null and *B*-null extremities, so we decrease *d* by 1 for a path, or by 2 for a cycle. Since the initial value of *d* is 2*n*, we end up with $d=d_{r}(A,B)$.

Algorithm 1 Algorithm to compute the distance between genomes *A* and *B*. Genome *A* is given as a list of length 2*n*, where *A*[*i*] = *j* if *Ae*_*i*_ = *e*_*j*_ , and *A*[*i*] = 0 if *Ae*_*i*_ = 0; similarly for *B*. The algorithm scores each component in *BG*(*A*,*B*) by comparing the numbers of *A*-null and *B*-null extremities. Equal numbers mean the component decreases the distance, by 1 for a path, or by 2 for a cycle.

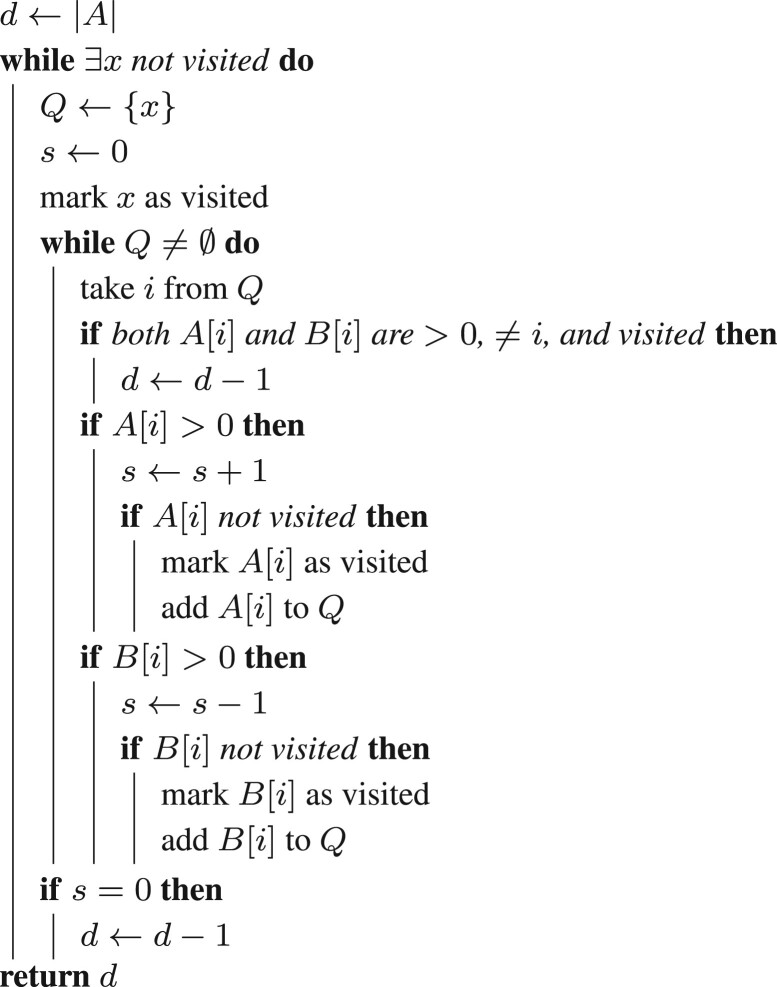

### 3.2 Basic operations

A matrix *X* is an *operation* when there is a genome *A* such that *A *+* X* is a genome. In this case, we say that *X* is *applicable* to *A*. The *weight* of an operation *X* is the rank of *X*. From here to the end of Section 3, we call these the *basic operations* to transform one genome into another when they do not share the same set of markers.

As expected, we need to consider the insertion or the deletion of an entire chromosome. Insertions and deletions of parts of chromosomes are not needed, as we show in the Supplementary Appendix. The matrix for the insertion or deletion of a chromosome with *k* markers is, up to the sign, equivalent to a genome with *k* markers, and always has weight 2*k*. Therefore, the weight of such an operation is 2*k*.

In addition, we consider a new kind of operation that takes advantage of our relaxed definition of genomes. Recall that, when we defined genomes in Section 2, we mentioned that, given a genome *A*, we do not require that $g_{t}\in V(A)$ if $g_{h}\in V(A)$, or *vice versa*. This relaxed definition now comes into play. We define an operation that substitutes a single extremity for an extremity that does not exist in the genome; due to its rank, we assign such an operation a weight of 2.

Introducing this kind of operation implies that the concept of chromosomes also has to be relaxed. In a genome where, for every g∈G, the extremities *g_h_* and *g_t_* are either both present or both absent, a chromosome is a sequence of markers that can be either circular, having no free ends, or linear, with exactly two free ends. In the case of a genome with only one extremity of a marker, there are *semi-chromosomes* that, instead of ending at a free end, end with an unpaired extremity, i.e. a head extremity whose corresponding tail is not in the genome, or *vice versa*. As a result, now an insertion or a deletion can be of a whole chromosome, or of a whole semi-chromosome, always with a weight equal to the number of extremities being inserted or deleted.

With the introduction of extremity substitutions, we now have six types of basic operations:

- Cut, with cost 1.
- Join, with cost 1.
- Double swap, with cost 2.
- Deletion of whole chromosomes or semi-chromosomes, costing the number of extremities deleted.
- Insertion of whole chromosomes or semi-chromosomes, costing the number of extremities inserted.
- Substitution of one extremity, with cost 2.

**Fig. 4.**
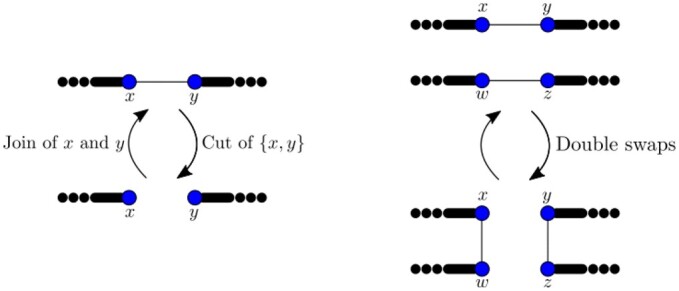
Examples of cuts, joins and double swaps

When the genomes considered have the same marker content, it was shown in previous work by the first and last authors along with P. Biller that only three types of operations are sufficient to sort any genome into another with respect to the rank distance, i.e. at a cost equal to the rank distance between them: cuts, joins and double swaps (Meidanis *et al.*, 2017). Cuts and joins have weight 1, while double swaps have weight 2. These operations are illustrated in Figure 4.

**Fig. 5.**
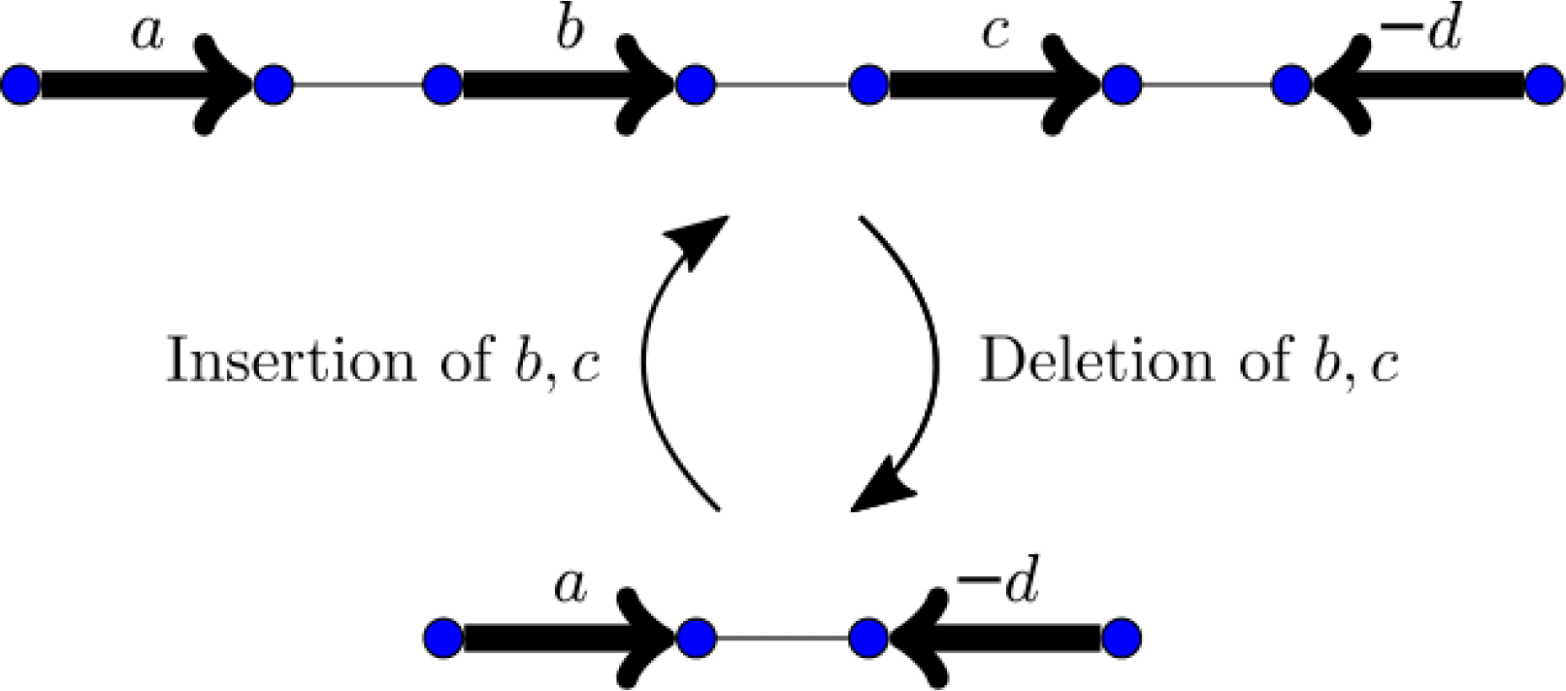
Example of an insertion of markers *b* and *c* and its inverse operation, the deletion of *b* and *c*

In this article, we seek to add to our model operations that deal with unequal gene content and, similarly to the work cited above on same-content genomes, to reduce them to a minimal sufficient set of basic operations. The first operations considered are insertions and deletions. These operations insert or delete contiguous blocks of markers, as in Figure 5.

The deletion of a contiguous section of *k* markers at the end of a chromosome has weight 2k+1. It is effectively equivalent to a cut separating these *k* markers from the rest of the chromosome, costing 1, followed by the deletion of the new chromosome, at a cost of 2*k*. If the deleted region is internal (does not include a free end), the deletion costs 2k+2. Such an operation is then equivalent to a double swap that extracts the region into a new circular chromosome followed by the deletion of this chromosome, also at a total cost of 2k+2.

A similar reasoning is valid for insertions, but in the inverse direction. Inserting a segment of *k* markers at the end of a chromosome is the same as inserting the *k* new markers as a linear chromosome and then applying a join between the new chromosome and its target. To insert a region inside a chromosome, we perform the insertion of a circular chromosome with the *k* markers, and then we use a double swap to incorporate the region into the chromosome. It is important to note that in the rank distance model, both linear and circular chromosomes can be inserted, and at the same cost per marker.

As a result, we can concern ourselves only with the deletion/insertion of whole chromosomes, as any other type of deletion can be replaced by a cut or double swap followed by a chromosome deletion, and insertions can be represented by a chromosome insertion plus a join or double swap. Therefore, we end up with a cast of five basic operations:

- Cuts or joins, with cost 1.
- Double swaps, with cost 2.
- Insertions or deletions of linear or circular chromosomes with *k* markers, with cost 2*k*.

Let *S* be the chromosome being inserted or deleted. Let *A*(*S*) be the set containing all the adjacencies {*x*, *y*} in *S*, and the singleton {*z*} for every free end *z* in *S*. Then, the deletion *D*(*S*) can be written as the matrix


$$D(S)=-\sum_{\left\{x,y\}\in A(S\right)}\left(xy^{t}+yx^{t}\right)-\sum_{\left\{x\}\in A(S\right)}xx^{t}.$$

On the other hand, the insertion of *S* can be written as the matrix −D(S). This covers both the case where *S* is circular and the case where it is linear.

**Fig. 6.**
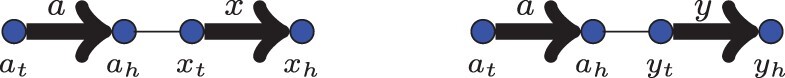
Example of two genomes that cannot be optimally sorted only with insertions and deletions. Left: genome *A*. Right: genome *B*. The distance *d*(*A*, *B*) is 4, but deleting the marker *x* and inserting *y* in its place would cost 6

The matrix for the insertion or deletion of a chromosome with *k* markers is, apart from the signs, equivalent to the matrix of a genome with *k* markers, and always has weight 2*k*. However, this initial set of basic operations is not sufficient to explain the changes in gene content under the rank distance. Consider the genomes in Figure 6. To go from *A* to *B* using only cuts, joins, double swaps, insertions and deletions, it would be necessary to cut the adjacency $\left\{a_{h},x_{t}\right\}$, delete *x*, insert *y* and join *a_h_* and *y_t_*. This sequence of operations would cost 6 (1 for the cut, 1 for the join, 2 for the deletion, and 2 for the insertion). Nevertheless, $d_{r}(A,B)=r(B-A)=4$.

This example shows that it is not enough to consider our initial set of basic operations. We thus introduce one more type of operation: the substitution. A substitution takes *p* contiguous markers, anywhere in the genome, and substitutes them with another block of *q* markers, at a cost of 2p+2q. Biologically, a substitution can be seen as the accumulation of a series of small mutations that transforms a block of markers into a block of different markers over time (Braga *et al.*, 2011b).

**Fig. 7.**
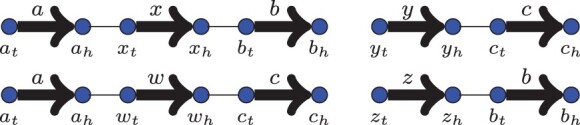
Example of two genomes that cannot be optimally sorted using only insertions, deletions and marker substitutions. Top: genome *A*. Bottom: genome *B*. The distance *d*(*A*, *B*) is 8, but substituting *x* for *w* and *y* for *z* already costs 8, and does not lead to genome *B*

Unfortunately, these substitutions are still not enough to sort genomes under the rank distance. Consider the genomes in Figure 7. The rank distance between them is eight, but there is no way to sort one into the other with the operations described so far, since just the two substitutions of *w* for *x* and *z* for *y* already cost 8, and do not move markers *b* and *c*.

One way of dealing with cases like this is to take advantage of our relaxed definition of genomes. Recall that when we defined genomes in Section 2, we mentioned that, given a genome *A*, we do not require that $g_{t}\in V(A)$ if $g_{h}\in V(A)$, or *vice versa*. This relaxed definition will now come into play.

Insertions, deletions and substitutions as described so far always act on both ends of every marker involved, either adding or removing the marker as a whole. But we may define an operation that substitutes a single extremity with another extremity that does not exist in the genome, and assign weight 2 to such an operation.

Introducing this kind of operation implies that the concept of chromosomes also has to be relaxed. In a genome where, for every g∈G, both extremities *g_h_* and *g_t_* are either present or absent, a chromosome is a sequence of markers that can be either circular, having no free ends, or linear, with exactly two free ends. In the case of a genome with only one extremity of a marker, there are *semi-chromosomes* which, instead of ending at a free end, end with an unpaired extremity, i.e. a head extremity whose corresponding tail is not in the genome, or *vice versa*. As a result, an insertion or a deletion can now be of a whole chromosome, or of a whole semi-chromosome, always with a weight equal to the number of extremities being inserted or deleted. With these more flexible definitions, the example in Figure 7 can be sorted with a total weight of 8, by performing four extremity substitutions: $x_{t}\to w_{t},x_{h}\to z_{h},y_{h}\to w_{h},y_{t}\to z_{t}$.

It may be hard to argue for the biological relevance of an event that replaces a single extremity, but mathematically they are capable of explaining the rank distance. With the introduction of extremity substitutions, we have now six types of basic operations:

- Cut, with cost 1.
- Join, with cost 1.
- Double swap, with cost 2.
- Deletion of whole chromosomes or semi-chromosomes, costing the number of extremities deleted.
- Insertion of whole chromosomes or semi-chromosomes, costing the number of extremities inserted.
- Substitution of one extremity for another, with cost 2.

As we explain next, it turns out that this collection of basic operations is sufficient to construct a scenario transforming any genome into any other genome, whether or not they have the same content, at a total cost equal to the rank distance between them.

### 3.3 Sorting and distance formula

Let $X=(X_{1},X_{2},\ldots ,X_{k})$ be a sequence of operations such that, for every 1≤i≤k, the operation *X_i_* is applicable to $A+X_{1}+\cdots +X_{i-1}$, and $A+X_{1}+\cdots +X_{k}=B$. We say that X is a *sorting scenario* from *A* to *B*. The *weight* of X is the sum of the ranks of its operations, i.e.


$$w(X)=\sum_{i=1}^{k}r(X_{i}).$$

We denote by *w*(*A*, *B*) the minimum weight of a sorting scenario from *A* to *B*. When a scenario X from *A* to *B* satisfies w(X)=w(A,B), we call X*optimal*. In Supplementary Appendix SB, we prove intermediate results to show that the rank distance *d*(*A*, *B*) is equal to the optimum weight of a scenario going from *A* to *B* using the basic operations listed in Section 3.2:


$$d_{r}(A,B)=w(A,B).$$

The following formula, proven in the Supplementary Appendix SA, allows for efficient (linear-time) computation of the rank distance, based on parameters of the augmented breakpoint graph:


$$d_{r}(A,B)=2n-2c(A,B)-p_{0}(A,B)-p_{AB}(A,B).$$

## 4 An alternative: the rank-indel distance

In order to avoid general extremity substitutions and genomes without both extremities of a marker, a different approach to the addition of indels to the rank distance is to define a genomic distance that includes the basic operations of the rank distance for genomes with the same content, plus insertions and deletions, all with the same weight as in the rank distance model. This way, we define the *rank-indel distance* $d_{i}(A,B)$ of *A* and *B* as the minimum cost of an operation sequence sorting *A* into *B*, using the basic operations:

- Cuts/joins, with cost 1.
- Double swaps, with cost 2.
- Insertions/deletions of linear or circular chromosomes with *k* markers, costing 2*k*.

We already know that $d_{i}(A,B)\geq r(B-A)$. This inequality can sometimes be strict. In fact, we obtain the following formula for the rank-indel distance between *A* and *B* (we prove it in Supplementary Appendix SC):


$$d_{i}(A,B)=2n-2c(A,B)-p_{0}(A,B)+p_{AB}(A,B).$$

## 5 Experiments

We ran experiments both on simulated and real data on a computer running Ubuntu version 16.04 with a 2.3 GHz AMD Ryzen 7 processor (eight cores) and 8 GB of RAM. Both comprise the computation of the DCJ-Indel, rank, and rank-indel distances between pairs of genomes from a set of genomes in order to construct distance matrices. For the DCJ-Indel distance, we used UniMoG (Braga *et al.*, 2011a), a software that unifies many rearrangement distance models, including the DCJ-Indel model. Although there exists a newer tool to compute the DCJ distance, namely DING, an ILP implementation developed by Bohnenkämper *et al.* (2021), we chose UniMoG because we do not use DING’s ability to handle repeated genes. In addition, DING is significantly slower than UniMoG because it leverages ILP to compute the DCJ distance. For the rank and rank-indel distances, we used in-house developed scripts available at https://github.com/meidanis-lab/rank-indel. We noticed that the results for rank and rank-indel distances were identical, which shows that the number of *AB*-null paths is small enough in practice to not result in differences reflected in a phylogenetic tree. Hence, we developed a single optimized script to compute the rank distance, which was used to obtain the results presented below.

After computing pairwise rearrangement distances between genomes and constructing distance matrices, we inferred phylogenetic trees using the Neighbor-Joining (NJ) algorithm (Saitou and Nei, 1987) as implemented in the ape package (Paradis and Schliep, 2019). The resulting trees were then compared against a reference tree using the Robinson–Foulds (RF) (Robinson and Foulds, 1981) and Quartet (Estabrook *et al.*, 1985) metrics, both implemented in the Quartet package (Brodal *et al.*, 2004; Sand *et al.*, 2014; Smith, 2019). The RF metric is a standard metric to compare phylogenetic trees, but we also adopted the Quartet distance because it outperforms a number of widely used tree distances, in particular, the RF metric, in some theoretical and practical metrics (Smith, 2020; Steel and Penny, 1993).

In Section 5.1, we describe the data analysis approach applied to a simulated dataset and a randomly generated phylogenetic tree, taken as the ground-truth. We do the same in Section 5.2, but for real bacterial genomes, *Escherichia coli* and *Shigella* species, and the phylogenetic tree constructed by Skippington and Ragan (2011) taken as our reference.

### 5.1 Performance benchmark

We generated a random phylogenetic tree, denoted hereafter as *T*, with 20 taxa using Ngesh (Tresoldi, 2021) with default parameters. This tree was fed to the simulation tool developed by Bohnenkämper *et al.* (2021), which samples gene order sequences over *T*. This tool starts from a random gene order sequence with user-defined length and samples Poisson-distributed DCJ events with expectation equal to the corresponding edge weights of *T*. The same applies to insertion, deletion and duplication events of one or more consecutive genes, but their frequency is dependent on a rate factor adjusted by the user. In addition, the length of each segmental insertion, deletion and duplication is drawn from a Zipf distribution, whose parameters are also adjusted by the user. We summarize the default values we used for the parameters in Table 2. In particular, the chosen values for the insertion and deletion rates are twice the values used by Bohnenkämper *et al.* (2021). We doubled this parameter in order to have a better assessment of the rank and DCJ-Indel distances when *indel* events occur more frequently. The duplication rate was set to 0 because we do not consider repeated markers. Using these parameters, the simulator outputs the leaf genomes of *T* as gene order sequences.

**Table 2.** Default parameters used in simulation tool

Parameter	No. of chromosomes	No. of genes	Insertion rate	Deletion rate	Duplication rate	Size of *indel*
Default value	20	5000	0.2	0.4	0	3.5

**Fig. 8.**
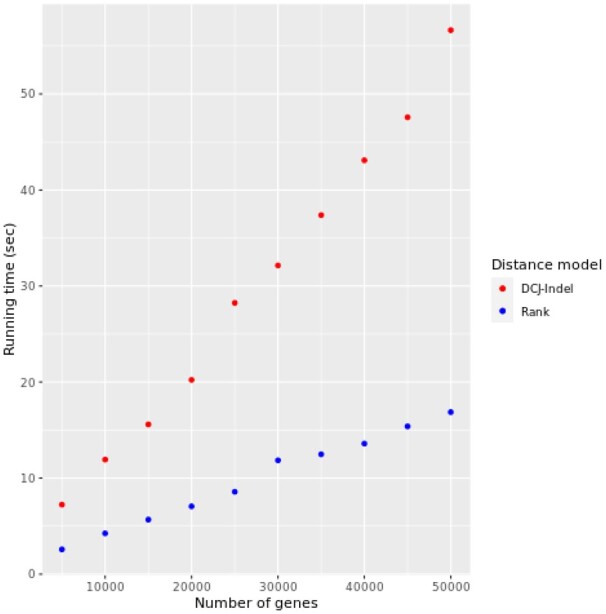
Running time of our implementation of the rank distance against DCJ-Indel as implemented in the UniMoG software package

To assess the performance of the rank and DCJ-Indel distances, we fixed the parameters to the values shown in Table 2, but varied the number of genes and insertion/deletion rates, one at a time. By varying the number of genes from 5000 to 50 000 in steps of 5000, we assessed the running time of our implementation of the rank distance and the DCJ-Indel distance as implemented in UniMoG. We note that UniMoG receives a single input file containing the markers of all genomes to be compared, similar to the FASTA format. It is unclear whether UniMoG uses parallelism in this computation. On the other hand, our implementation of the rank distance receives two input files, each containing the markers of a single genome. This allowed us to compute the rank distance between pairs of genomes in parallel using eight cores. Figure 8 shows the running time of the rank and DCJ-Indel implementations as a function of the number of genes. Note that the curves reflect the linear-time complexity of both distance models.

**Fig. 9.**
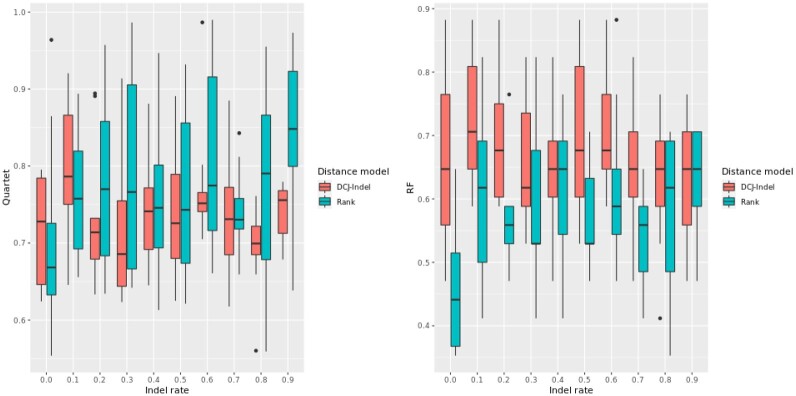
Each box plot corresponds to 10 phylogenetic trees compared against the ground-truth; the insertion and deletion rates were varied and the remaining parameters were fixed. Left: Quartet metric. Right: normalized RF metric

In the second experiment, we varied both the insertion and deletion rates from 0.0 to 0.9 in steps of 0.1. For each step, we inferred 10 phylogenetic trees for each distance model, compared each one against the ground-truth using the Quartet and normalized RF metrics, and generated box plots. The results are shown in Figure 9.

We observe that the rank distance, on average, outperforms the DCJ-Indel distance in the Quartet metric, even though the rank distance exhibits greater variability for this metric. As for the normalized RF metric, the similarity of the resulting trees with the ground-truth remains stable between 60% and 70% under the DCJ-Indel distance, on average, whereas the rank distance shows comparable results only for higher rates of *indel* events; for lower rates of *indel* events, the results for the rank distance are mixed and overall inconclusive. This discrepancy may be due to the fact that the RF metric is more sensitive to the relocation of taxa, whereas the Quartet metric is more stable (Smith, 2020; Steel and Penny, 1993). We conclude that the rank distance is more robust to higher rates of *indel* events, or, equivalently, to genomes that greatly differ in their marker content. This may indicate that the rank distance can provide better approximations when comparing genomes of distantly related species, while for closely related species both distance models are equally applicable if one uses the Quartet metric as a comparator.

### 5.2 Real data analysis

We used the phylogenetic tree for the *E.coli* and *Shigella* species constructed by Skippington and Ragan (2011) as our reference. The *E.coli* strains were divided into five distinct *E.coli* reference collection phylogenetic groups—A, B1, B2, D and E—while the *Shingella* strains were grouped under the S group. That tree has 27 taxa, but not all genomes were available for download at the National Center for Biotechnology Information (NCBI). Hence, we used a subset of the tree—with 20 genomes in total—described in that study and restructured the reference tree accordingly. Table 3 lists the genomes, we used.

**Table 3.** Data downloaded from NCBI used in our study

Accession	Species	Strain	Identifier
GCF002949755.1	*Shigella dysenteriae*	07-3308	SD_073308
GCF000013585.1	*Shigella flexneri*	8401	SF_8401
GCF000007405.1	*Shigella flexneri*	2457 T	SF_2457T
GCF000020185.1	*Shigella boydii*	BS512; CDC 3083-94	SB_CDC308
GCF000007445.1	*Escherichia coli*	CFT073	EC_CFT073
GCF003028775.1	*Escherichia coli*	E24377A	EC_24377A
GCF000010245.2	*Escherichia coli*	K-12 substr. W3110	EC_K12W31
GCF000017765.1	*Escherichia coli*	HS	EC_HS
GCF000013305.1	*Escherichia coli*	536	EC_536
GCF000013265.1	*Escherichia coli*	UTI89	EC_UTI89
GCF000010385.1	*Escherichia coli*	SE11	EC_SE11
GCF000019645.1	*Escherichia coli*	SMS-3-5	EC_SMS35
GCF000019385.1	*Escherichia coli*	ATCC 8739	EC_ATCC87
GCF000026265.1	*Escherichia coli*	IAI1	EC_IAI1
GCF000026285.1	*Escherichia coli*	S88	EC_S88
GCF000026245.1	*Escherichia coli*	55989	EC_55989
GCF000026325.1	*Escherichia coli*	UMN026	EC_UMN026
GCF000026345.1	*Escherichia coli*	IAI39	EC_IAI39
GCF000026305.1	*Escherichia coli*	ED1a	EC_ED1a
GCF000026225.1	*Escherichia fergusonii*	ATCC 35469 T	EF_ATCC35

*Note*: The ‘Identifier’ column lists the labels used in the phylogenetic trees.

**Fig. 10.**
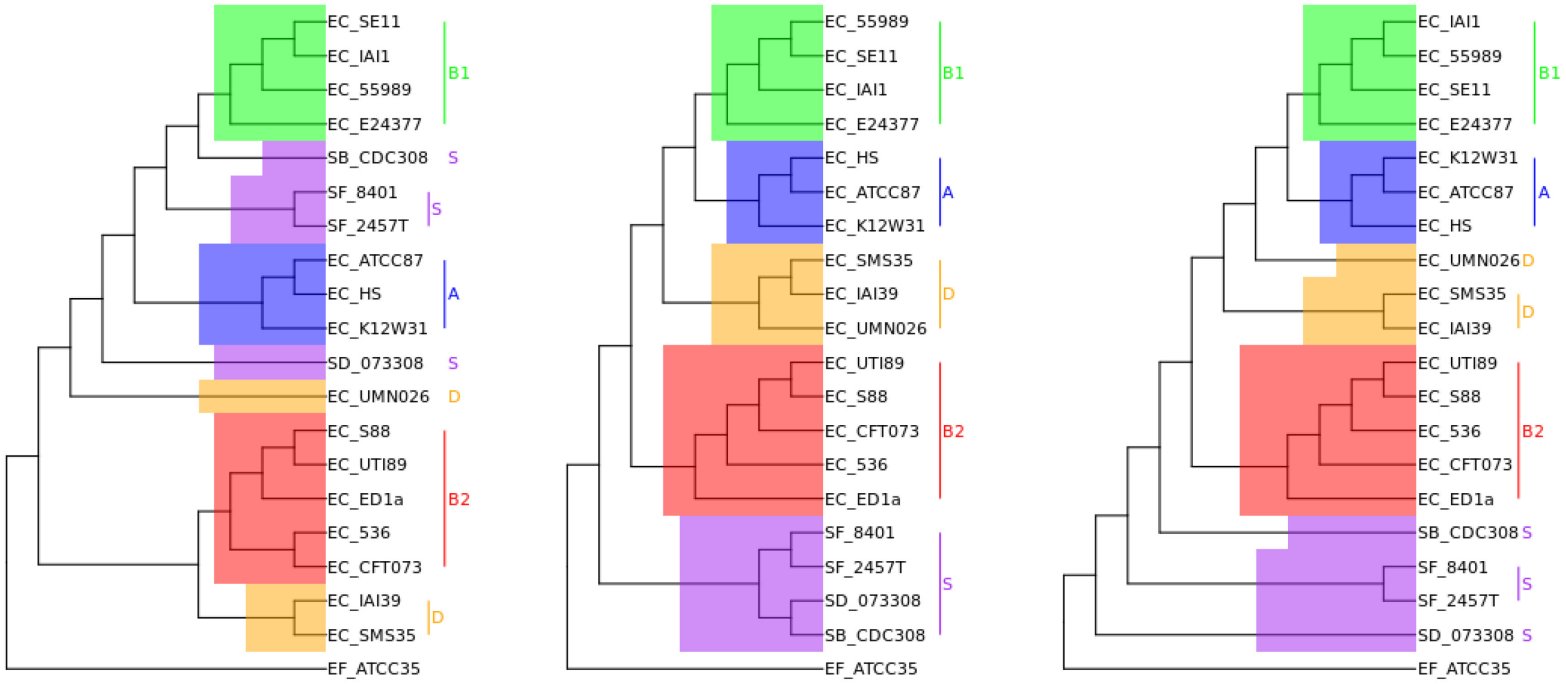
Left: subset of reference tree constructed by Skippington and Ragan (2011). Middle: Rank/Rank-Indel tree. Right: DCJ-indel tree

We could maintain all the groups, except the *E.coli* strains of group E. Following Skippington and Ragan (2011) and Touchon *et al.* (2009), our reference tree is rooted using *Escherichia fergusonii* as an outgroup. The restructured reference tree is shown in Figure 10. Note that group D is the only one reported as polyphyletic, while groups A, B1 and B2 are monophyletic. In addition, *Shigella dysenteriae* appears as an ancestor of the other *Shigella* strains of group S.

In this experiment, markers are genes and we need a way to ascertain gene homology. We used NCBI’s Protein Clusters as follows. Most annotated genomes from NCBI contain non-redundant protein record (WP) identifiers, which can be mapped to a protein cluster. Genes are considered homologous if, and only if, they map to the same cluster.

We wrote scripts to download generic feature format (GFF) files from NCBI corresponding to the genomes and extracted the WP accessions for all their genes. Most of the GFF files had WP accessions; those that did not were discarded. The next step was to use the mapping from WP to the Protein Cluster Database provided by NCBI to operate on this translation. Genes mapped in this manner served as our comprehensive marker set G in this analysis. Any paralogous genes (duplicated genes in the same genome) were discarded. At the end of this filtering process, each genome consisted of a sequence of genes along with their orientations. We ran pairwise comparisons between the genomes using the rank, rank-indel and DCJ-Indel distances to construct distance matrices and infer phylogenetic trees with the NJ algorithm. Finally, we compared our resulting trees against the reference tree using the Quartet and normalized RF metrics. The resulting metrics are shown in Table 4. Note that the metrics for the rank and rank-indel distances were the same; indeed, the computed phylogenetic trees for both rank distances were identical. Figure 10 shows the resulting trees for the rank and DCJ-Indel distances.

**Table 4.** RF and Quartet distances from our trees against the reference

	Rearrangement distance		
Metric	Rank	Rank-Indel	DCJ-Indel
Robinson–Foulds	0.470	0.470	0.411
Quartet	0.656	0.656	0.657

The rank distances clustered all groups as monophyletic, whereas the DCJ-Indel distance placed groups D and S as polyphyletic. Although these groups are also polyphyletic in the reference tree, the placement of these strains within each group—specially group S of the *Shigella* strains—is very different between the two trees. The *Shigella* and *E.coli* species are closely related and challenging to differentiate at the sequence level (Chattaway *et al.*, 2017; Devanga Ragupathi *et al.*, 2018). On the other hand, whole-genome-based approaches show that they are distinct species, although sister species within the *Escherichia* genus (Zuo *et al.*, 2013). This is concordant with our results, which show that *Shigella* and *E.coli* can be distinguished at the genome level just by looking at the order and orientation of genes. Moreover, the rearrangement-based distances placed the *Shigella* species as the most distant relative of the *E.coli* strains, a feature not seen in the reference tree.

Overall, the rank and DCJ-Indel distances exhibited comparable results for this set of bacterial genomes, differing most in the placement of groups D and S. Although we conclude that both distances are adequate for phylogenetic inference, further studies are needed to assess their applicability in evolutionary molecular biology, since our experiments are restricted to prokaryotic genomes of well-known species.

## 6 Conclusion

In this article, we expanded the rank distance to account for genomes with different gene content, but still without duplications. The first step, in Section 2, was to define genomes that do not necessarily contain all the markers of G. This allows for the representation of genomes with different markers from each other, and is done very naturally, by using zeros in the rows/columns corresponding to the missing markers. We then developed two ways to compare these genomes.

The first approach simply extends the rank distance, keeping the distance $d_{r}(A,B)$ between two genomes *A* and *B* equal to the rank r(B−A) of their difference. We showed how to efficiently compute *d_r_*, and how to transform *A* into *B* using only basic operations, adding insertions, deletions and the substitution of a single extremity to the cast of basic operations of the rank distance of genomes with the same markers.

The substitution of single extremities leads to genomes with incomplete markers. To avoid this, we also present an alternative rank-indel distance that changes the content of a genome only through insertions and deletions of chromosomes. We note that both distances have very simple formulas, and are closely related, with $d_{i}(A,B)=d_{r}(A,B)+2p_{AB}(A,B)$.

We conducted experiments with real and simulated data using the rank, rank-indel and DCJ-indel distances. We noted that the phylogenetic trees for the rank and rank-indel distances were the same, showing that the distinguishing term in their formulas is very small in our experiments. In all cases, the phylogenetic trees constructed from real data were able to cluster related species within their respective clades, although the topological comparison against the reference tree shows many differences. A notable feature of the rearrangement-based phylogenetic trees is the clear distinction between the *Shigella* and *E.coli* species. In this regard, the rank and rank-indel distances exhibit similar results when compared to the DCJ-indel distance. As for the experiments with simulated data, we observed that the rank distance outperformed the DCJ-Indel distance in the Quartet metric, which is more robust than the RF metric for phylogenetic tree comparisons (Smith, 2020; Steel and Penny, 1993). This may indicate that the rank distance is a better model for multi-chromosomal genomes with unequal marker content, but still without repeated markers. Recall that both DCJ and rank represent different extensions of the algebraic distance (Meidanis and Dias, 2000)—originally defined for circular chromosomes—to linear chromosomes. They differ in the way they count the number *N* of genes after circularization of linear paths: the DCJ model takes the maximum *N* between the two genomes being compared, while rank takes the average *N* (Meidanis and Yancopoulos, 2013).

Overall, we noted that there is enough phylogenetic signals in the order and orientation of genes alone, which makes rearrangement-based distances applicable to phylogenetic studies. Moreover, the rank and DCJ distance models are, to the best of our knowledge, the only rearrangement-based distances capable of handling multi-chromosomal genomes with linear and circular chromosomes, which makes these distances the most applicable from the genome rearrangement literature. Although sequence-based methods are the gold standard in phylogenetics, rearrangement-based methods are faster and can provide good approximations. Nevertheless, further studies are needed to better assess the usefulness of these distances in wider biological contexts, e.g. inferring the evolutionary history of eukaryotic genomes. Lastly, although we introduced the notion of a semi-chromosome, which may seem absurd from a biological perspective, this article contributed further evidence that modeling genome evolution with the rank distance has biological relevance and can be used in practice.

## Supplementary Material

btad087_Supplementary_Data

## Data Availability

The data underlying this article are available in the National Center for Biotechnology Information (NCBI) FTP site at https://ftp.ncbi.nlm.nih.gov/, and can be accessed with unique identifiers contained in the GitHub repository mentioned in the Abstract, directory config, file esche_shige.csv. *Conflict of Interest*: none declared.
