# Supplementary material for: Generalizations of the genomic rank distance to indels
Source: Bioinformatics. 2023 Feb 15;39(3):btad087. doi: 10.1093/bioinformatics/btad087 (PMC9985151; doi:10.1093/bioinformatics/btad087)
Supplement: btad087_Supplementary_Data [file btad087_supplementary_data.pdf]

A Proofs for Section 3.1 — Efficient Computation

Throughout this section, let A and B be two genomes. We seek to efficiently compute $d_r(A, B) = r(B - A)$. Although genomic matrices in this model are not necessarily invertible, they share some properties with invertible matrices, as shown in the next lemma.

Lemma 1. *If A is a genome and x and y are extremities such that $Ax = y$, then $Ay = x$. In particular, if $Ax \neq 0$, then $AAx = x$.*

Proof. If $Ax = y$, then either $x \neq y$ and $\{x, y\}$ is an adjacency in A, or $x = y$ and x is a free end in A. Therefore, in either case, $Ay = x$. By combining the two statements, we get $A(Ax) = Ay = x$. □

Now, let us define a relation between extremities. Given two extremities x and y, $x \sim_{AB} y$ if there exists an integer $k \geq 0$ such that $x = (AB)^k y$ or $x = (BA)^k y$. Similarly to the relation defined between extremities in [1], this is an equivalence relation. We will omit the subscript and simply write $x \sim y$ when the genomes are clear from the context.

- $x \sim x$ because $x = (AB)^0 x$
- $x \sim y$ implies $y \sim x$ because, by a repeated application of Lemma 1, if $x = (AB)^k y$, then $y = (BA)^k x$, and vice-versa.
- $x \sim y$ and $y \sim z$ implies $x \sim z$. If $x = (AB)^k y$ and $y = (AB)^j z$, then $x = (AB)^{k+j} z$. The same goes for the case where $x = (BA)^k y$ and $y = (BA)^j z$. If $x = (AB)^k y$ and $y = (BA)^j z$, we can use Lemma 1 to write $y = (BA)^k x = (BA)^j z$. Assuming $k \leq j$ without loss of generality, we get

$$(BA)^k x = (BA)^{k+(j-k)} z \implies$$
$$\implies x = (BA)^{j-k} z,$$

  where the simplification follows from a repeated application of Lemma 1. The remaining case, where $x = (BA)^k y$ and $y = (AB)^j z$, is proven analogously.

We call the classes of this equivalence relation *AB-orbits*. Given a set of extremities S, we denote by $\chi(S)$ its characteristic vector in the standard basis:

$$\chi(S) := \sum_{x \in S} x.$$

Given an orbit S, if $A\chi(S) = B\chi(S)$, we call S a *balanced orbit*; otherwise, S is *unbalanced*. Balanced orbits can be used to construct a basis of $\ker(A - B)$.

Lemma 2. *Let x and y be two arbitrary extremities. If $x \sim y$, then $x - y \in$ $\mathrm{im}(A - B)$.*

Proof. Let $x = (AB)^k y$ for $k \geq 0$. We will prove the lemma by induction on k.

The base case is when $k = 0$, i.e., $x = y$. Then, we have $x - y = 0$, which belongs to $\mathrm{im}(A - B)$.

Now, suppose that $(AB)^k y - y \in \mathrm{im}(A - B)$ for all y and some $k \geq 0$, and let $x = (AB)^{k+1} y$. Define $z = (AB)^k y$. By the induction hypothesis, $z - y \in \mathrm{im}(A - B)$. Additionally, $x = ABz$. Since $Bz \neq 0$, we can use Lemma 1 to write

$$\begin{aligned} x - z &= ABz - BBz \\ &= (A - B)Bz. \end{aligned}$$

And therefore $x - z \in \mathrm{im}(A - B)$. Finally, $x - y = (x - z) + (z - y) \in \mathrm{im}(A - B)$. □

The following result classifies the various types of connected components in the augmented breakpoint graph according to the orbits they contain.

Lemma 3. *Consider the augmented breakpoint graph $BG(A, B)$ of genomes A and B. Each connected component in $BG(A, B)$ corresponds to one or two AB-orbits, as follows:*

1. *A cycle contains two orbits, both balanced.*
2. *If a path is proper, A-null, or B-null, it contains a single orbit, which is balanced when the path is proper, and unbalanced otherwise.*
3. *An AB-null path contains two orbits, one balanced and one unbalanced.*
4. *An AA or BB-null path contains two orbits, both unbalanced.*

Proof.
1. A cycle in $BG(A, B)$ must have an even number of vertices because its edges alternate between edges in A and edges in B. In a cycle $v_1, v_2, \ldots, v_{2k}$, the mapping AB corresponds to walking two steps in one direction, while BA corresponds to walking two steps in the other direction. This means that the odd-numbered vertices are all equivalent to one another, as are all the even-numbered vertices. Since the cycle is even, no odd-numbered vertex is equivalent to an even-numbered vertex. Therefore, we end up with two orbits: $\{v_1, v_3, \ldots, v_{2k-1}\}$ and $\{v_2, v_4, \ldots, v_{2k}\}$. Notice also that

$$A(v_1 + v_3 + \ldots + v_{2k-1}) = B(v_1 + v_3 + \ldots + v_{2k-1}) = v_2 + v_4 + \ldots + v_{2k}$$

and

$$A(v_2 + v_4 + \ldots + v_{2k}) = B(v_2 + v_4 + \ldots + v_{2k}) = v_1 + v_3 + \ldots + v_{2k-1},$$

showing that each orbit is balanced.
2. In a path $v_1, v_2, \ldots, v_k$, as in the case of a cycle above, all the odd-numbered vertices are pairwise equivalent, as are all the even-numbered vertices. However, if there is a free end in the path, and there are at least two vertices, this free end is equivalent to its neighbor, making all the vertices in the path equivalent. If the path consists of a single vertex, then it is clearly a singleton

orbit. In both cases, we have a single orbit. If the path is proper and has at least two vertices, then

$$A(v_1 + v_2 + \ldots + v_k) = B(v_1 + v_2 + \ldots + v_k) = v_1 + v_2 + \ldots + v_k,$$

so the orbit is balanced. A proper path with only one vertex also gives rise to a balanced orbit, because either $A(v_1) = B(v_1) = v_1$ if v_1 is free in both A and B, or $A(v_1) = B(v_1) = 0$ if v_1 is null in both A and B. On the other hand, if the path is A-null or B-null and e is the null vertex, then

$$e^t A(v_1 + v_2 + \ldots + v_k) \neq e^t B(v_1 + v_2 + \ldots + v_k),$$

since one of these expressions is zero and the other isn't, showing that the orbit cannot be balanced.

3. An AB-null path $v_1, v_2, \ldots, v_{2k+1}$ has at least two vertices, an even number of edges, and therefore an odd number of vertices. As in the previous cases, the odd-numbered vertices are pairwise equivalent, as are the even-numbered ones. In this case, however, since there are no free ends, these two sets of vertices constitute separate orbits. Notice that

$$A(v_1 + v_3 + \ldots + v_{2k+1}) = B(v_1 + v_3 + \ldots + v_{2k+1}) = v_2 + v_4 + \ldots + v_{2k},$$

so the odd-numbered vertices form a balanced orbit. On the other hand,

$$v_1^t A(v_2 + v_4 + \ldots + v_{2k}) \neq v_1^t B(v_2 + v_4 + \ldots + v_{2k}),$$

since one side of this equation is zero while the other isn't, showing that the even-numbered vertices form an unbalanced orbit.

4. An AA or BB-null path $v_1, v_2, \ldots, v_{2k}$ has an even number of vertices. As in the previous case, the odd-numbered vertices form an orbit, and the even-numbered ones form a distinct orbit, since there are no free ends. Both are unbalanced, since

$$v_1^t A(v_2 + v_4 + \ldots + v_{2k}) \neq v_1^t B(v_2 + v_4 + \ldots + v_{2k}),$$

because one side is zero and the other isn't, and also

$$v_{2k}^t A(v_1 + v_3 + \ldots + v_{2k-1}) \neq v_{2k}^t B(v_1 + v_3 + \ldots + v_{2k-1}),$$

for a similar reason.

□

We want to show now that the set $\mathcal{K}$ of all vectors $\chi(S)$ such that S is a balanced orbit forms a basis for $\ker(A - B)$. To do so, we need to show that:

- For every $v \in \mathcal{K}$, we have $(A - B)v = 0$. This follows directly from the definition of balanced orbits.
- $\mathcal{K}$ is linearly independent. This comes from the fact that each extremity is present in at most one vector of $\mathcal{K}$ (the vectors in $\mathcal{K}$ have disjoint supports).

– $\mathcal{K}$ generates $\ker(A-B)$. This will be proven below.

Lemma 4. *Let e be an extremity such that $Ae = 0$. Then, for every $v \in \ker(A-B)$, we have $(Be)^t v = 0$.*

Proof. We have

$$(Be)^t v = e^t Bv = e^t Av = (Ae)^t v = 0^t v = 0.$$

□

Lemma 5 (Lemma 6 of [1]). *If $v \in \ker(A-B)$ and $x \sim y$, then $x^t v = y^t v$.*

Proof. From Lemma 2, we know that $x - y \in \operatorname{im}(A-B)$. Since $\operatorname{im}(A-B)$ and $\ker(A-B)$ are orthogonal due to the symmetry of $A-B$, we have $(x-y)^t v = 0$, and therefore $x^t v = y^t v$. □

Lemma 6. *If S is an unbalanced orbit, there is an extremity $e \in S$ such that either Ae or Be is a null extremity.*

Proof. According to Lemma 3, all unbalanced orbits come from null paths. If S comes from an A-null or B-null path $v_1, v_2, \ldots, v_k$, then $S = \{v_1, v_2, \ldots, v_k\}$. Assume, without loss of generality, that v_1 is the null extremity in the path. Then either $Av_2 = v_1$ or $Bv_2 = v_1$.

If S comes from AB-null path $v_1, v_2, \ldots, v_{2k-1}$, then $S = \{v_2, v_4, \ldots, v_{2k-2}\}$. Both v_2 and v_{2k-2} are adjacent to a null extremity in one of the genomes.

If S comes from an AA-null path $v_1, v_2, \ldots, v_{2k}$, then $S = \{v_1, v_3, \ldots, v_{2k-1}\}$ or $S = \{v_2, v_4, \ldots, v_{2k}\}$. Both orbits also satisfy the lemma, because $Bv_{2k-1} = v_{2k}$ and $Bv_2 = v_1$. A similar reasoning applies to the case of a BB-null path. Since there are no other cases of null paths, the lemma is proved. □

Lemma 7. *The set $\mathcal{K}$ generates the kernel of $A-B$.*

Proof. According to Lemma 5, any $v \in \ker(A-B)$ can be written as

$$v = \sum_i c_i \chi(S_i),$$

where the S_i are the disjoint AB-orbits.

If S_i is an unbalanced orbit, Lemma 6 states that there is an extremity $e \in S_i$ such that either Ae or Be is a null extremity. For this e, by Lemma 4, we have $e^t v = 0$, and, consequently, $c_i = 0$.

Therefore, v is a linear combination of vectors $\chi(S)$, where S is a balanced orbit. □

With Lemma 7, we conclude that the dimension of $\ker(A-B)$ is equal to the number of balanced orbits, and, consequently, we can state the following:

Theorem 8.

$$d_r(A,B) = 2n - 2c(A,B) - p_0(A,B) - p_{AB}(A,B).$$

Proof. By counting the number of balanced orbits present in each type of component, according to Lemma 3, we get

$$\dim \ker(A - B) = 2c(A, B) + p_0(A, B) + p_{AB}(A, B),$$

and the desired result follows immediately from the rank-nullity theorem. □

B Proofs for Section 3.3 — Sorting

We now show that the rank distance $d(A, B)$ is equal to the optimum weight of a scenario going from A to B using the basic operations listed in Section 3.2.

Lemma 9. *Given two genomes A and B, we have*

$$d_r(A, B) \leq w(A, B).$$

Proof. Let $\mathcal{X} = X_1, X_2, \ldots, X_k$ be a scenario such that $w(\mathcal{X}) = w(A, B)$. Repeatedly applying the triangle inequality to intermediate genomes of the form $A + X_1 + \ldots + X_i$, we have

$$d_r(A, B) \leq \sum_{i=1}^{k} d_r(A + \sum_{j=1}^{i-1} X_j, A + \sum_{j=1}^{i} X_j).$$

However,

$$d_r(A + \sum_{j=1}^{i-1} X_j, A + \sum_{j=1}^{i} X_j) = r(A + \sum_{j=1}^{i-1} X_j - (A + \sum_{j=1}^{i} X_j)) = r(X_i).$$

Therefore,

$$d_r(A, C) \leq \sum_{i=1}^{k} r(X_i) = w(\mathcal{X}) = w(A, C).$$

□

We say an operation X on genome A is *sorting* with respect to genome B when $d_r(A + X, B) = d_r(A, B) - r(X)$. Similarly, an operation Y on genome B is *sorting* with respect to genome A when $d_r(B + Y, A) = d_r(A, B) - r(Y)$. When A and B are fixed, we say that an operation is sorting if it falls into one or the other of these categories.

We say a component of $BG(A, B)$ is *sorted* if it is a proper 0-path or a 2-cycle, that is, a path with 0 edges or a cycle with 2 edges. The relevance of sorted components stems from the fact that when all the components of the breakpoint graph $BG(A, B)$ are sorted, we have $A = B$. Therefore, one strategy to transform A into B is to sort component by component of the breakpoint graph. This is the approach we take here. In addition, we consider sorting operations in both directions (applied to A and sorting with respect to B, or vice versa), because

all the basic operations we consider have inverses that are themselves basic. Furthermore, for any genome X, a sequence of operations $\mathcal{X}$ starting from A ending up at X and a sequence of operations $\mathcal{Y}$ starting from B ending up at X can be combined into $\mathcal{Z} := \mathcal{X} \circ -\mathcal{Y}^R$, where $-\mathcal{Y}^R$ contains the inverses of the operations in $\mathcal{Y}$ listed in reverse order, and $\circ$ denotes list concatenation.

Lemma 10. *If $Ax \neq 0$, $Ax \neq x$, and $Bx = x$, then cutting the adjacency $\{x, Ax\}$ in A is always sorting.*

Proof. In the breakpoint graph $BG(A, B)$, the node corresponding to the extremity x is the end of a path. Let P be this path. Let X be the cut of adjacency $\{x, Ax\}$. The graph $BG(A+X, B)$ has the same components as $BG(A, B)$, except for P. Instead of P, there are two paths. The first is a path with all the nodes of P except for x. It has the same type as P. The second is a proper 0-path with node x. Therefore, $d_r(A+X, B) = d_r(A, X) - 1$, because the number of proper paths increases, while n, c, and p_{AB} remain the same. □

Lemma 11. *If $BG(A, B)$ has at least one path with at least 3 edges, or one cycle with at least 4 edges, there is a sorting double swap.*

Proof. In either case, we can take two edges from the same genome, with one edge from the other genome incident to both, to define a double swap. For the cycle, this double swap splits the cycle into two smaller ones. For the path, this double swap transforms the path into a cycle and a path of the same type as the original path. In both cases, the number c of cycles increases by 1, decreasing the distance by 2. □

Lemma 12. *If $BG(A, B)$ has at least one AB-null 2-path, there is a sorting extremity substitution.*

Proof. Let x and y be the A-null and the B-null ends of a 2-path P, respectively. Let X be the operation that substitutes y with x. The graph $BG(A+X, B)$ has the same components as $BG(A, B)$, except for P. Instead of P, there is a 2-cycle containing x and Bx, and a proper 0-path with y. Thus, $d_r(A+X, B) = d_r(A, X)+1-3 = d_r(A, X)-2$, because we gain an extra cycle, and an AB-path is replaced by a proper path. □

Lemma 13. *If $BG(A, B)$ is only composed of sorted components plus AA-null and BB-null paths of length 1, and A-null and B-null paths of length 0, then A can be sorted into B using only sorting insertions and deletions.*

Proof. Without loss of generality, suppose there are no AB-null 0-paths. With this extra assumption, there are $|V(A) \cap V(B)|$ nodes in the sorted components, and $|V(A) \setminus V(B)| + |V(B) \setminus V(A)|$ in the remaining components. The distance between A and B is

$$\begin{aligned} d_r(A, B) &= 2n - |V(A) \cap V(B)| \\ &= |V(A) \setminus V(B)| + |V(B) \setminus V(A)|. \end{aligned}$$

Add all the A-null nodes to A, also joining all ends of AA-null paths. These additions are materialized by a number of insertions on A, at a total cost of $|V(B) \setminus V(A)|$. The edges between the ends of the AA-null paths are adjacencies in the inserted chromosomes, so they do not add extra cost.

Similarly, remove all B-null nodes from A, removing the edges of the BB-null paths at the same time. These whole-chromosome deletions cost a total of $|V(A) \setminus V(B)|$ units. Let A' be the genome created by the application of these operations.

These operations transformed all the AA-null and BB-null paths into proper 0-paths and 2-cycles, making all the components in $BG(A', B)$ sorted. This means $d_r(A', B) = 0$, and therefore $A' = B$. Hence, this procedure sorted A into B at the cost of $d_r(A, B)$. □

Theorem 14. *Given two genomes A and B,*

$$d_r(A, B) = w(A, B).$$

Proof. Lemmas 10—13 give us an outline for a sorting algorithm:

1. Apply cuts to A and B until both genomes have the same free ends;
2. Apply double swaps until the only remaining components are sorted ones, as well as AB-null 2-paths, and AA-null and BB-null paths of length 0 or 1;
3. Apply substitutions until there are no AB-null 2-paths left;
4. Apply the necessary insertions or deletions to make both genomes equal.

Let $\mathcal{X}$ be a sorting scenario obtained with the procedure above. Because all operations in $\mathcal{X}$ are sorting, and reduce the distance by exactly their weight, we have $w(\mathcal{X}) = d_r(A, B)$. Since $w(A, B) \leq w(\mathcal{X})$, by Lemma 9, $d_r(A, B) = w(A, B)$. □

C Proofs for Section 4 — An Alternative: the Rank-Indel Distance

We prove this formula in the remainder of this section. To begin with, let

$$f(A, B) = 2n - 2c(A, B) - p_0(A, B) + p_{AB}(A, B).$$

Given an operation X applicable to A, define $\Delta f(A, B; X)$ as follows:

$$\Delta f(A, B; X) = f(A + X, B) - f(A, B).$$

Similarly, given a statistic $s(A, B)$ of $BG(A, B)$ (that is, s can be one of c, p, p_0, p_{AA}, etc.), define

$$\Delta s(A, B; X) = s(A + X, B) - s(A, B).$$

We also denote by $u(A, B)$ the *number of unique markers* between A and B:

$$u(A, B) = (|V(A) \setminus V(B)| + |V(B) \setminus V(A)|)/2.$$

Lemma 15. *If operation X is the insertion of a chromosome with k markers into genome A and $B = A + X$, then $\Delta f(A, B; X) \geq -2k$.*

Proof. The $2k$ inserted extremities are all A-null ends of paths in $BG(A, B)$. Therefore,

$$\Delta p_A(A, B; X) + 2\Delta p_{AA}(A, B; X) + \Delta p_{AB}(A, B; X) = -2k,$$

with each term being non-positive. No proper path is affected by the insertion, so

$$\Delta p_0(A, B; X) = 0.$$

The number of cycles can only increase via AA-null paths being closed, so

$$\Delta c(A, B; X) = -\Delta p_{AA}(A, B; X).$$

Therefore, we can write

$$\begin{aligned}\Delta f(A, B; X) &= 2n - 2c(A + X, B) - p_0(A + X, B) + p_{AB}(A + X, B)\\ &\quad -(2n - 2c(A, B) - p_0(A, B) + p_{AB}(A, B))\\ &= -2\Delta c(A, B; X) - \Delta p_0(A, B; X) + \Delta p_{AB}(A, B; X)\\ &= 2\Delta p_{AA}(A, B; X) + \Delta p_{AB}(A, B; X)\\ &\geq -2k.\end{aligned}$$

□

Lemma 16. *If operation X is the deletion of a chromosome with k markers from genome A and $B = A + X$, then $\Delta f(A, B; X) \geq -2k$.*

Proof. Since the deletion is of an entire chromosome of B-null extremities, the only adjacencies affected are those between two B-null extremities. Therefore, the only components that undergo changes are BB-null paths of length 1, each of them turning into two single nodes absent in both genomes (which we defined to be proper paths), and 0-length B-null paths that turn into 0-length (also proper) paths. Thus,

$$\Delta p_0(A, B; X) \leq 2k$$

and

$$\Delta p_{AB}(A, B; X) = \Delta c(A, B; X) = 0.$$

Therefore, we can write

$$\Delta f(A, B; X) = -2\Delta c(A, B; X) - \Delta p_0(A, B; X) + \Delta p_{AB}(A, B; X) \geq -2k.$$

□

Lemma 17. $d_i(A, B) \geq 2n - 2c(A, B) - p_0(A, B) + p_{AB}(A, B)$.

Proof. Notice that the right hand side is $f(A,B)$. Let $X_1, X_2, \ldots, X_m$ be an optimal sequence of operations sorting A into B. Define $A_0 = A$, $A_1 = A_0 + X_1$, $A_2 = A_1 + X_2$, and so on, so that $A_m = B$. In order to show that the inequality $d_i(A,B) \geq f(A,B)$ holds, we will first prove that no operation can cause a change in the formula greater than its weight, that is, for any integer i such that $1 \leq i \leq m$, we want to show that

$$-\mathit{\Delta} f(A_{i-1}, B; X_i) \leq w(X_i).$$

This will be done by an exhaustive case analysis. We examine all options for the operation X_i and its impact on the breakpoint graph. Note that, by definition, $\mathit{\Delta} f(A,B;X) = \mathit{\Delta} p_{AB}(A,B;X) - 2\mathit{\Delta} c(A,B;X) - \mathit{\Delta} p_0(A,B;X)$.

- X_i is a cut, so $w(X_i) = 1$
  - If X_i cuts an edge in a cycle, the cycle turns into a proper path, so $\mathit{\Delta} f(A_{i-1}, B; X_i) = 1$.
  - If X_i cuts an edge in an AA-null, BB-null, or AB-null path, this path turns into two paths, each of them A-null or B-null, so $\mathit{\Delta} f(A_{i-1}, B; X_i) = 0$ in the first two cases and $\mathit{\Delta} f(A_{i-1}, B; X_i) = -1$ in the third case.
  - If X_i cuts an edge in any other type of path, it turns into a path of the same type, and one proper path, and $\mathit{\Delta} f(A_{i-1}, B; X_i) = 1$.

  Notice that in all these cases, $-\mathit{\Delta} f(A_{i-1}, B; X_i) \leq w(X_i)$.
- X_i is a join, so $w(X_i) = 1$

  Recall that only free ends can be joined, and note that the free ends being joined must be present in both genomes.
  - A join of two proper paths generates one proper path, so $\mathit{\Delta} f(A_{i-1}, B; X_i) = 1$.
  - A join of a proper path and an A-null or B-null path generates an A-null or B-null path, so $\mathit{\Delta} f(A_{i-1}, B; X_i) = 1$.
  - A join of two A-null or B-null paths generates an AA-null, BB-null, or AB-null path, so $\mathit{\Delta} f(A_{i-1}, B; X_i) = 0$ in the first two cases and $\mathit{\Delta} f(A_{i-1}, B; X_i) = 1$ in the third case.
  - A join of the two ends of a proper path generates a cycle, so $\mathit{\Delta} f(A,B;X) = -1$.

  In all these cases, we have indeed $-\mathit{\Delta} f(A_{i-1}, B; X_i) \leq w(X_i)$.
- X_i is a double swap, so $w(X_i) = 2$
  - A double swap involving two proper paths results in two other proper paths, so $\mathit{\Delta} f(A_{i-1}, B; X_i) = 0$.
  - A double swap involving a proper and an A-null or B-null path results in another proper path and an A-null or B-null path, so $\mathit{\Delta} f(A_{i-1}, B; X_i) = 0$.
  - A double swap involving a proper and an AA-null, BB-null or AB-null path results in two paths, each of which is A-null or B-null, so $\mathit{\Delta} f(A_{i-1}, B; X_i) = 0$ or $\mathit{\Delta} f(A_{i-1}, B; X_i) = -1$.
  - A double swap involving two A-null or B-null paths results in two other A-null or B-null paths, or a proper path together with an AA-null, BB-null or AB-null path, so $\mathit{\Delta} f(A_{i-1}, B; X_i) = 0$ or $\mathit{\Delta} f(A_{i-1}, B; X_i) = -1$.

- A double swap involving an A-null or B-null path on the one hand, and an AA-null, BB-null or AB-null path on the other hand, results in one A-null path or a B-null path and one AA-null, BB-null or AB-null path, so $-1 \leq \Delta f(A_{i-1}, B; X_i) \leq 1$.
- A double swap involving two AA-null or BB-null paths results in two AA-null or BB-null paths, or two AB-null paths, so $\Delta f(A_{i-1}, B; X_i) = 0$ or $\Delta f(A_{i-1}, B; X_i) = 2$.
- A double swap involving two AB-null paths results in an AA-null path and a BB-null path, or two AB-null paths, so $\Delta f(A_{i-1}, B; X_i) = -2$ or $\Delta f(A_{i-1}, B; X_i) = 0$.
- A double swap involving a path and a cycle results in a longer path of the same type, so $\Delta f(A_{i-1}, B; X_i) = 2$.
- A double swap applied to two cycles results in a longer cycle, in which case $\Delta f(A_{i-1}, B; X_i) = 2$.
- A double swap applied to two edges of the same path either results in a path of the same type with a reversed segment, in which case $\Delta f(A_{i-1}, B; X_i) = 0$, or a path of the same type and a cycle, in which case $\Delta f(A_{i-1}, B; X_i) = -2$.
- A double swap applied to two edges of the same cycle either results in another cycle, in which case $\Delta f(A_{i-1}, B; X_i) = 0$, or in two shorter cycles, in which case $\Delta f(A_{i-1}, B; X_i) = -2$.

We conclude that in all these cases we always have $-\Delta f(A_{i-1}, B; X_i) \leq w(X_i)$.

– X_i is an insertion of k markers, so $w(X_i) = 2k$
In this case, our result follows immediately from Lemma 15.

– X_i is a deletion of k markers, so $w(X_i) = 2k$
In this case, our result follows immediately from Lemma 16.

Now we can reason as follows. The expression $f(A, B)$ can be written as a summation of deltas:

$$\begin{aligned}
\sum_{i=1}^{m}(-\Delta f(A_{i-1}, B; X_i)) &= \sum_{i=1}^{m}(-f(A_{i-1} + X_i, B) + f(A_{i-1}, B)) \\
&= -f(A_1, B) + f(A_0, B) \\
&\quad -f(A_2, B) + f(A_1, B) \\
&\quad -f(A_3, B) + f(A_2, B) \\
&\quad \ldots \\
&\quad -f(A_m, B) + f(A_{m-1}, B) \\
&= f(A_0, B) - f(A_m, B) \\
&= f(A, B) - f(B, B) = f(A, B).
\end{aligned}$$

Then we can use this fact to conclude:

$$\begin{aligned} 2n - 2c(A,B) - p_0(A,B) + p_{AB}(A,B) &= f(A,B) \\ &= \sum_{i=1}^{m} (-\Delta f(A_{i-1}, B; X_i)) \\ &\leq \sum_{i=1}^{m} w(X_i) \\ &= d_i(A,B). \end{aligned}$$

□

There is a simple way to equalize the gene content of A and B. Examining $BG(A,B)$, add the A-null nodes to A, and the B-null nodes to B. Join the ends of any AA-null (BB-null) path in A (B), respectively. This process generates genomes A^* and B^* such that $V(A^*) = V(B^*) = V(A) \cup V(B)$. We call A^* and B^* the *augmented genomes* of A and B.

Lemma 18. *$d_i(A, A^*) + d_i(B, B^*) \leq 2u(A,B)$.*

Proof. The addition of the A-null nodes to A is realized by a number of insertions on A, at a total cost of $|V(B) \setminus V(A)|$. The edges between the ends of the AA-null paths are adjacencies in the inserted chromosomes, so they do not add extra cost to the construction of A^*.

Similar insertions in B form B^*, at a cost of $|V(A) \setminus V(B)|$. Therefore, $d_i(A, A^*) + d_i(B, B^*) \leq 2u(A,B)$. □

Since A^* and B^* have the same gene content, we already know how to sort them.

Lemma 19. *Let A and B be two genomes without duplications. If $BG(A,B)$ has c cycles, p paths, and d AA-null or BB-null paths, then*

$$d_i(A^*, B^*) = 2n - 2c(A,B) - p_0(A,B) - 2u(A,B) + p_{AB}(A,B).$$

Proof. The breakpoint graph $BG(A^*, B^*)$ has $c(A,B) + p_{AA}(A,B) + p_{BB}(A,B)$ cycles, and $p_0(A,B) + p_A(A,B) + p_B(A,B) + p_{AB}(A,B)$ paths, all proper, and therefore

$$\begin{aligned} d_i(A^*, B^*) = 2n &- 2(c(A,B) + p_{AA}(A,B) + p_{BB}(A,B)) \\ &- (p_0(A,B) + p_A(A,B) + p_B(A,B) + p_{AB}(A,B)). \end{aligned}$$

Taking into account that

$$2u(A,B) = p_A(A,B) + p_B(A,B) + 2p_{AA}(A,B) + 2p_{BB}(A,B) + 2p_{AB}(A,B),$$

we can write

$$d_i(A^*, B^*) = 2n - 2c(A,B) - p_0(A,B) - 2u(A,B) + p_{AB}(A,B).$$

□

Lemmas 18 and 19, together with the triangle inequality, which necessarily holds for any distance defined via a set of allowed operations with non-negative weights, give us a lower bound on the indel distance $d_i(A, B)$.

Corollary 20. $d_i(A, B) \leq 2n - 2c(A, B) - p_0(A, B) + p_{AB}(A, B)$.

Combining Lemma 17 with this last corollary we have our result:

Theorem 21. *Given genomes A and B,*

$$d_i(A, B) = 2n - 2c(A, B) - p_0(A, B) + p_{AB}(A, B).$$

References

1. João Meidanis, Priscila Biller, and João Paulo Pereira Zanetti. A Matrix-Based Theory for Genome Rearrangements. Technical Report IC-17-11, Institute of Computing, University of Campinas, August 2017. In English, 45 pages.
